# Assessment of risk factors on construction projects in gondar city, Ethiopia

**DOI:** 10.1016/j.heliyon.2022.e11726

**Published:** 2022-11-17

**Authors:** Amare Tilahun Tessema, Getachew Asefa Alene, Natnael Melsew Wolelaw

**Affiliations:** a Transportation Engineering, Department of Civil Engineering, Debre Tabor University, Debre Tabor, Ethiopia; b Structural Engineering, Department of Civil Engineering, Debre Tabor University, Debre Tabor, Ethiopia; c Road and Transport Engineering, Department of Civil Engineering, Debre Tabor University, Debre Tabor, Ethiopia

**Keywords:** Risk factors, Rank, Risk allocation, Modelling, Construction industry, Ethiopia

## Abstract

Construction projects are incredibly dynamic, which means a lot of dangers that could impact the success of the project in Gondar, Ethiopia. Therefore, study aimed to identify the risk factors affecting project performance, and also develop the research model representing risk factors affecting project performance. Data was collected from 26 valid responses were collected via questionnaire survey from the construction professionals. The stratified simple random sampling technique is used in the present study. A total of 26 completed and valid questionnaires were returned, yielding an 81.25 percent response rate. Factor analysis (FA) was employed to extract the factors of construction projects where seventeen independent factors (risk factors) and one dependent factor (project performance) were extracted. SPSS-23 software was used to analyze the relationship among three risk factors and their impact on project performance. The results showed that the five top-ranked risk variables with the highest magnitude of impacts, based on a quantitative risk analysis using the RII method and risk allocation number, were inflation and price increases; flawed design; poor material quality; delayed payment to the contractor, and subpar work. The five risk variables that were least important and had the least impact were labor strikes, a lack of a clear scope of work, delays in acquiring site access, and a lack of site access. Factor analysis and regression modeling were employed to determine the importance of the risk factors. The eight most significant risk variables for building projects were determined using the results of the factor analysis, and they are as follows: Construction and design risk, poor management, insufficient funding, uncertain political conditions, a lack of economics in the project budget, a lack of law and order, an unfavorable climate, and other risk factors are just a few examples of risk factors. The regression model indicates that inflation and price rises, delays in acquiring site access, and late contractor payments have a significant impact on the project's overall risk factors. The construction sector in Gondar, Ethiopia is anticipated to benefit significantly from these findings in terms of managing risk elements in construction contracts.

## 1 Introduction

Many development policies in many countries aim to promote the construction industry in order to prevent companies from collapsing unexpectedly [1]. The construction industry's growth necessitates an understanding of risk management principles. Every stage of the building process, from the initial investment appraisal to the construction and operation of the completed facility, is fraught with risk for all parties involved. Risk and uncertainty have the potential to be detrimental to construction projects [2]. Even though risk is inherent in all project endeavors, it is widely recognized that risk can be properly managed to limit its negative effects on project objectives. As a result, risk analysis and management are still an important part of construction project management nowadays in order to cope effectively with uncertainty and unexpected events and achieve project success. Risk management helps key project participants client, contractor or developer, consultant, and supplier in meeting their obligations and minimizing negative effects on construction project performance in terms of cost, time, and quality [3].

Basically, risk management isn't a new concept, and it's been used for a long-time using professionals' opinions [4]. Risk management in a construction project incorporates identifying inducing factors that could potentially negatively impact a project's cost schedule or quality baselines; measuring the associated possible effect of the identified risk; and applying actions to manage and alleviate the possible effect [3].

The construction industry in Ethiopia is challenged by several problems and thus making efforts in developing the construction industry is very difficult and complex [5]. Because of the effects on quality, time, and cost of building projects, the nature, occurrence, and impact of risk in construction have recently become a topic of concern [6].

The rapid growth of the Ethiopian economy necessitates enormous infrastructure and asset development. While this provides opportunities for project stakeholders, it is critical to employ effective risk management methods to deal with risks associated with variable construction activities in order to complete projects on time, on budget, and with high quality, safety, and environmental sustainability.

Several authors' previous research focused on identifying and categorizing risks associated with construction projects. Some of the authors include [7, 8, 9, 10, 11, 12], and others mentioned numerous risk factors that contributed to a project suspension, cost overruns, and quality deprivation. They all identified several risk variables and categorized them into internal and external hazards that could arise during construction projects.

From the works of the above scholars, external risks that are beyond the project team's control were classified as environmental risk, political risks, economic risks, legal risks, social risks, and nature risks. On the other hand, internal risks that arise from the specific nature of the project and events and are within the control of the project team were divided into design risks, financial risk, construction risks, management risks. In this research, both external and internal risks, as well as the categories that correspond with them, are identified and used for the assessment of risk management practice in the construction of building projects in Gondar city.

Risk can be defined as an uncertain event or condition that, if it occurs, has a positive or a negative effect on a project objective. According to [13]risk is defined as the exposure to loss/gain, or the probability of occurrence of loss/gain multiplied by its respective magnitude.

### 1.1 Factors of risk

According to the [14], the typical risk on a construction project is as: occurrence of accidents, failure to complete within the specified time, failure to obtain the expected outline, unforeseen adverse conditions, unexpected rises for labor and materials, force majeure, and failure to complete the project within the client's budget.

According to [15] presented 122 risk factors identified in construction projects which have been categorized into fifteen risk classifications. The risk identification can be done in various ways. Generally, the risks are classified into two [16].

#### 1.1.1 External risk

External risks are not directly related to the construction process, but they have a significant weight in terms of the completion of the project. They can be categorized as follows.

##### 1.1.1.1 Environmental risk

Climate change has an adverse effect on projects all around the world. Although environmental risk cannot be eliminated, it can be decreased by taking the required actions. Environmental risk plays a significant role in large building projects, notably transportation projects. To avoid material durability being reduced owing to climatic change, construction supplies should be appropriately stocked. During the construction material selection process, the material that is most suited to the site location and climatic circumstances should be consider [17].

##### 1.1.1.2 Political risk

Political risk definitions can be divided into two categories [18]. The first approach focused on the causes of political risk, such as the possibility of negative consequences from political events (such as wars, regime changes, revolutions, political violence, riots, insurgencies, terrorist attacks, and coups) and government activities (e.g., expropriations, unfair compensations, foreign exchange restrictions, illegal interferences, changes in laws, corruption, poor enforcement of the contract, and labor restrictions) [19]. The second approach focused on the repercussions of political risk, which was characterized as negative effects on businesses as a result of a deterioration in the political environment [20].

##### 1.1.1.3 Economic risk

In terms of economic risk, inflation and unexpected price fluctuations are the most significant [21]. Currency fluctuations are an economic risk to consider when designing a significant construction project, especially for international enterprises. Many countries have recently used foreign cash to build privately financed infrastructure, putting their local currencies in danger of depreciation. International lenders are rarely willing to take such a risk, preferring to get payments in foreign currency. In the past, public corporations or governments were willing to absorb currency risk; but now, with the increased need for private financing, the risk of currency depreciation now falls mostly on the project promoter, and the lender is unwilling to assume it [22]. This risk factor refers to challenges or concerns about the project's macroeconomic impact on the community and region in which it will be built [23].

##### 1.1.1.4 Legal and order

Some of the legal and order-related risks include project partner breach of contract, lack of enforcement of legal judgment, improper verification of contract documents, lack of knowledge of arbitration, and uncertainty and unfairness of court justice [17].

#### 1.1.2 Internal risk

Internal risks in large building projects are frequently linked to the management team's control. They can be categorized as follows.

##### 1.1.2.1 Design risk

Defects in the design that cause the asset to be built yet fail to meet the specified standards, legal requirements, and any environmental or other constraints. Such situations necessitate a revision to the project, resulting in delays and, most importantly, cost increases. Defects or failures in the design that cause the project to fall short of the contract's service standards, or that cause a rise in operation and maintenance (O & M) expenses to fulfill the contract's service requirements.

Some design risks include design flaws and omissions, the design process taking longer than expected, stakeholders requesting late revisions, and failing to complete the work in line with the contract [24].

##### 1.1.2.2 Technical risk

Anything that prevents from creating a product that fits a customer's desire is considered a technical risk. This can be due to a lack of funding and materials, a lack of site assessment, or an imperfect design. Changes in project scope and needs, as well as design flaws or omissions, are major causes of these risks.

Material shortage, poor quality of procured material, unknown site physical condition, Obsoleteness of building equipment, wastage of materials by work. are also some of the technical risks [17].

##### 1.1.2.3 Construction risk

Contractors and subcontractors are more likely to be the sources of most construction risks. Experienced contractors must be involved in the project as early as possible to make sound preparations for generating legitimate construction programs in order to keep the construction activity on schedule. Construction phase risks include machinery, delays due to rain and other factors, uncertain market conditions, contractor productivity issues, time [24]. Construction risks also include labor productivity, labor disputes, site conditions, equipment failures, design revisions, too high-quality standards, and new technologies [25].

##### 1.1.2.4 Management risk

The wrong project team selection, lack of a proper project manual, lack of procedures, a project being too complex for the available resources, inability to take prompt remedial actions, poor quality control, poor control of status review, lack of an experienced person in the project team, and inadequate communication infrastructure are some of the construction management risks.

Short tendering time, improper project feasibility study, time constraint, and no past experience in similar projects, are also some of the management risks [17].

##### 1.1.2.5 Finance risk

The ability to overcome the project's financial risk all the way to ultimate completion and operation is referred to as “financial risk.” This risk factor refers to challenges or concerns about the project's funding, such as the execution period, operations, or equity financing [23].

### 1.2 Risk management process

A typical risk management process includes risk identification, risk assessment, risk mitigation and risk monitoring [26]

### 1.3 Risk identification

**Figure 1:**
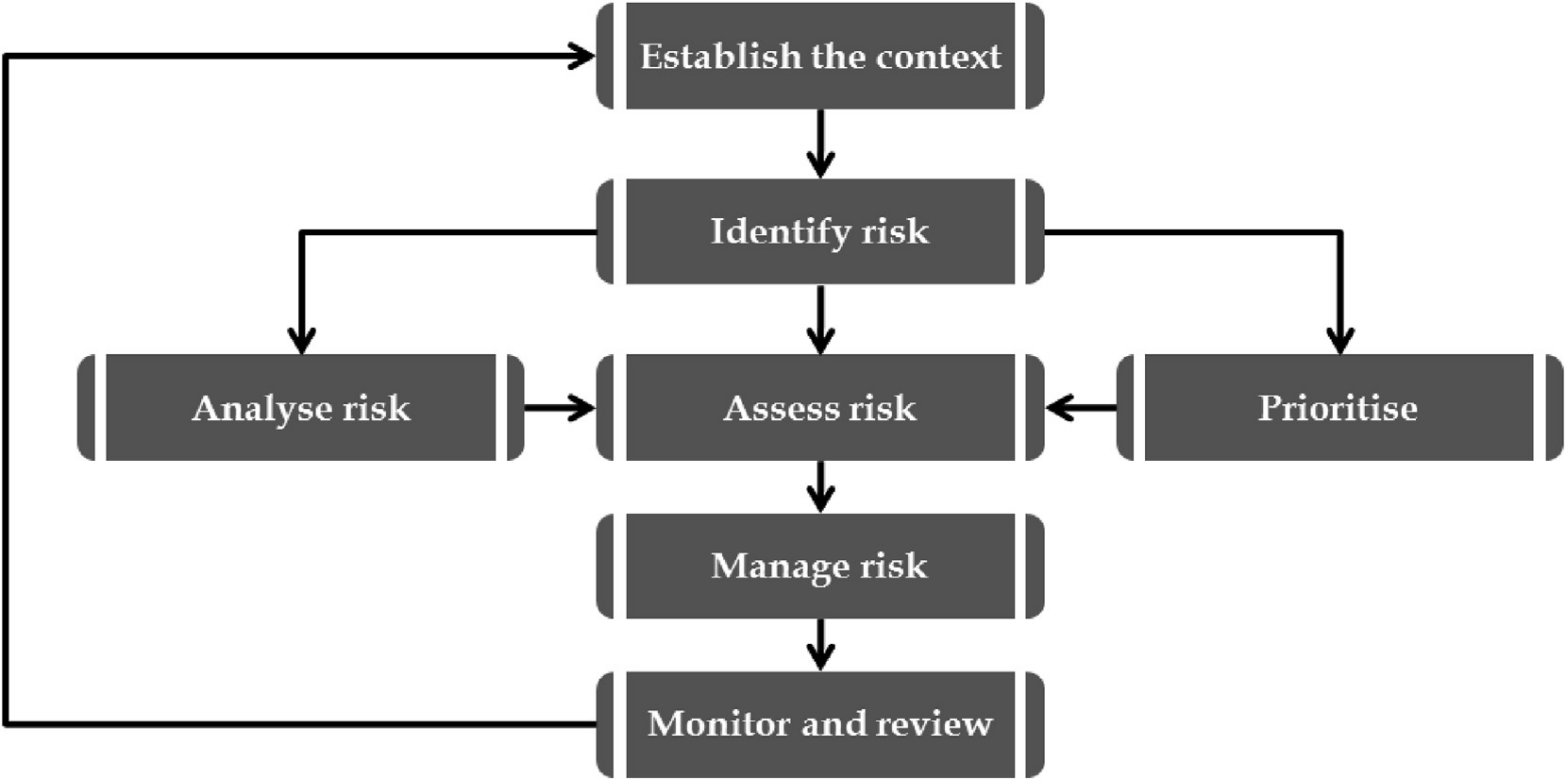
The process of risk management [26].

From Figure 1, the first step in the risk management process is risk identification, which involves identifying and examining potential risks and risk sources associated with construction projects [27, 28]. Several studies have been done to identify and classify construction risk variables [29] classified 3 risk types and identified them in 39 individual risks factors, referring to some as activity risks that may affect individual activities, while others were global risks that were common to all activities. Identifying the source and nature of risks is the first step in risk identification. Risk identification involves identifying the source and type of risks. According to [14], the detected risk is no longer a risk but rather a management issue.

### 1.4 Risk allocation

According to [30] the contractor and client perspectives that the top risk factors faced by construction industry are as awarded design to unqualified designers, accepted defective design, occurrence of accidents because of poor safety procedures, difficulty to access the site (vary far, settlement), inaccurate quantities, lack of consistency between bill of quantities, drawing and specification, working a hot (dangerous) areas, financial failure of the contractor, and high competition in bids. Contract provisions can simply be used to transfer or share them from one party to another [31]. agree, stating that contractors should be prepared to take a certain level of risk as a result of unplanned construction expenses, and that risk is also a concern for clients. The risk management approach includes such a risk allocation.

After the literature review, the findings of the study will contribute to risk factors in the Ethiopian construction sector, specifically in Gondar city, as well as provide useful information to international companies interested in providing construction project management services to Gondar city and the country of Ethiopia. The aims of this study to identify the risk factors affecting project performance, and develop the research model representing risk factors affecting project performance.

## 2 Research methodology

**Figure 2:**
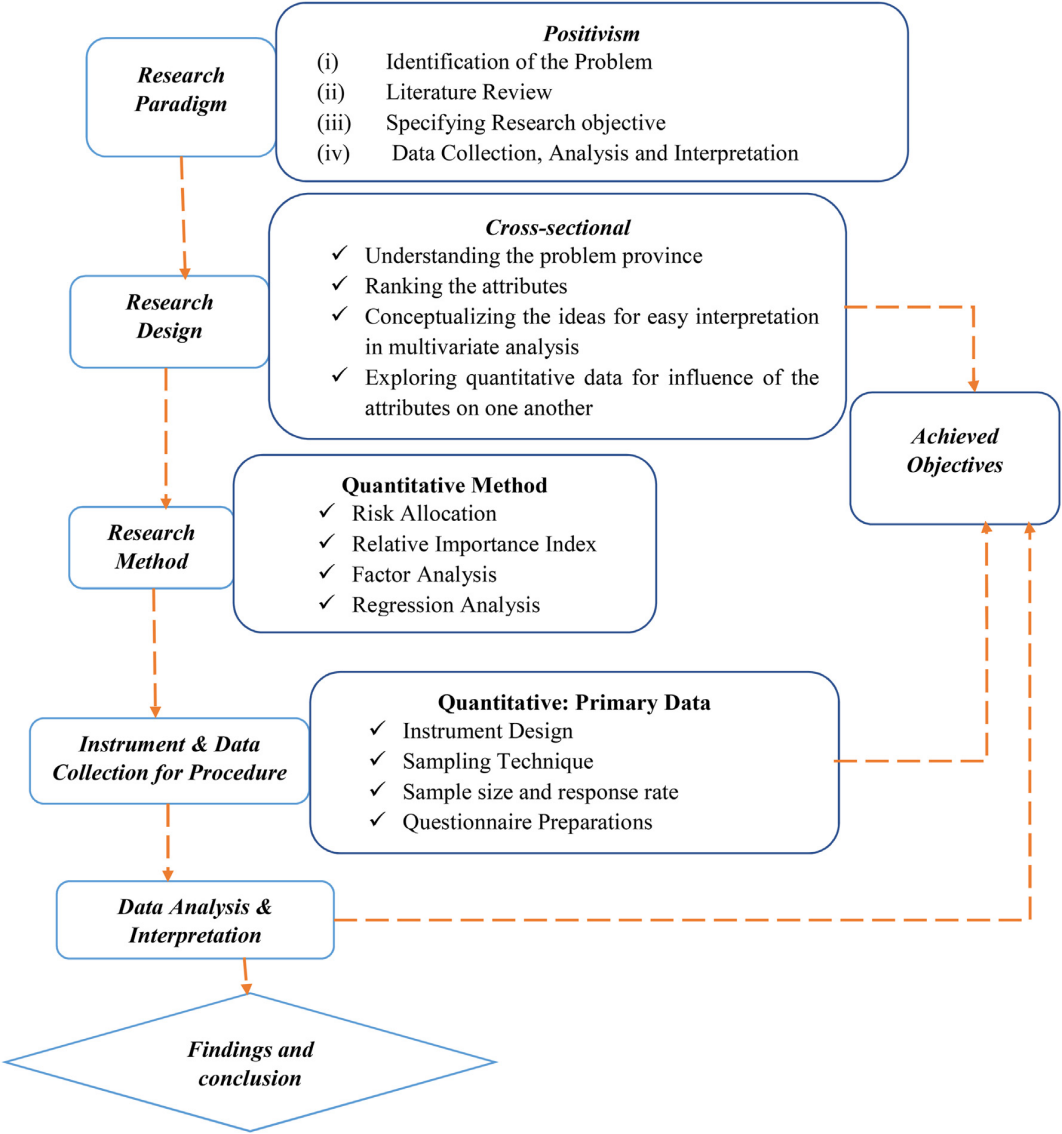
Flow chart of Research Methodology.

This section discusses the research methodology that was employed to accomplish the study's research objectives. The initial section of the research report is the methodological flow chart, which is depicted in Figure 2. It contains details about the research design, research method/approach, sampling technique, sample size and response rate, tools and data collection methods. Appropriate approaches have been adapted and used in the present study in order to illustrate the clarifications of these instruments. Lastly, this section demonstrates the appropriate statistical techniques used to analyze the data in this study.

### 2.1 Research design

The descriptive method is employed in this study in order to examine how risk factors affect building projects in Gondar, Ethiopia, among construction businesses. However, determining the level of risk factors associated with building in Gondar, Ethiopian construction companies, is a descriptive type of research. In order to establish correlations between predictors and criterion variables, field studies aim to build relationships between predictors and things that are causal [32, 33]. The study used a standardized questionnaire that was given to the chosen building construction specialists in the research area. While there are other ways of collecting data, such as interviews and observations, questionnaires are the most effective method when the researcher is confident of exactly what is needed and how to quantify the variables of interest [34].

### 2.2 Research method

The primary risk indicators in the context of the construction companies in Gondar, Ethiopia, have been identified using a quantitative technique. Data on the measured attributes was collected by a questionnaire survey. The respondents were required to select one of the projects in which they had participated and then react affirmatively to a question asking them to rate the perceived impact of the measurable attributes. A five-point Likert scale (1-very insignificant, 2- insignificant, 3-fairly significant, 4- significant, and 5-very significant) was adopted for guiding the participants to provide their objective responses with varying degrees of agreement or disagreement.

There were two sections to the questionnaire, though. The respondents' demographic information was gathered in section-1. Section-2 of the study included an evaluation of the impact magnitude of 21 risk variables for sustainable construction practices that were taken from the literature. On a 5-point Likert scale, the respondents were asked to express their views regarding the extent to which they would have an adverse effect on the use of sustainable construction techniques. Thus, this study used a quantitative methodology and primary data collected through a structured questionnaire.

### 2.3 Sampling technique

Currently, more than 20 construction sites were found in Gondar town, northwest Ethiopia and the major sites are owned by eight different companies (Amhara Water Works Enterprise, Nigldu Kibrit, Unity Engineering, 3M Construction, Midroc Construction, Afrotsion Construction, Alemayehu Ketema Construction and Aielmi Construction). Sampling can be thought of as a research technique for selecting an appropriate study subject from the population [35]. The stratified simple random sampling technique is used in the present study. The sample size was proportionally distributed because each site has a different number of occupations as shown in Table 1. Finally, the professionals in the registration book were used as a sampling frame to select the study subjects from each site using a stratified simple random selection technique.

**Table 1:** Respondents profile rate.

Stakeholders	Parties in the construction industry			Total in each category	% of role of profession
	Client	Contractor	Consultant		
Office Engineer	3	2	2	7	27
Architect	1	0	3	4	15
Project Manager	1	3	0	4	15
Site Engineer	3	2	1	6	23
General Manager	1	1	3	5	19
Total	9	8	9	26	
% by parties	35	31	35		

### 2.4 Sample size and response rate

The population of this study consisted of all pertinent registered building industry experts who have worked for at least ten years and were found on the registers of several professional organizations in Gondar City, Northwest, Ethiopia. Experience with the topic among the respondents is crucial in this type of research to prevent any accusations of bias. It is also thought that professionals with at least ten years of relevant experience may be able to provide the needed details about the risks involved in implementing sustainable construction practices, as they may have worked on a variety of building projects that should have given them enough knowledge about the dangers involved with such projects. Table 1 shows a brief description of respondents' profile rate in terms of professional role and stakeholders who participated in the study. A total of 32 questionnaires were distributed by hard copy, out of which 26 valid responses were obtained with a response rate of 81.25%. Moreover, response rate of 81.25%, that is acceptable as per [36], as they state that a minimum response rate of 50% can be taken as adequate. Response frequency of these questionnaires in this area of research differs, for instance [37], 51% [38], 43%; and in [39] 44%. So, in the present study, a response rate of 81.25% is good enough to make further analysis of collected data and to get more accurate results. Amongst the respondents, the highest proportion (27%) was from the office engineer involved in construction activities followed by the site engineer (23%). Respondents from the roles of general managers and both architects and project managers were 19% and 15% respectively.

### 2.5 Instruments and procedures of data collection

A standardized questionnaire and observational checklists were used to collect the data. With modifications to address contextual difficulties linked to the study environment and participants, the data collection procedures were adapted from the literature reviews [40, 41, 42]that had been published and were similar to this study. The questionnaire asked questions about demographic information about the respondents and comprised critical questions to which respondents were asked to respond. The tools were pretested on workers who weren't really included in the study but shared characteristics with the study subjects in a separate town, and required corrections were made. Data collectors and supervisors received instruction on data collection methods and equipment. The checklists' components were things like working environment and safety procedures. Before the data collectors moved on to the next interview, we verified the data right away and made any necessary corrections.

### 2.6 Instrument for data analysis

#### 2.6.1 Risk allocation

**Figure 3:**
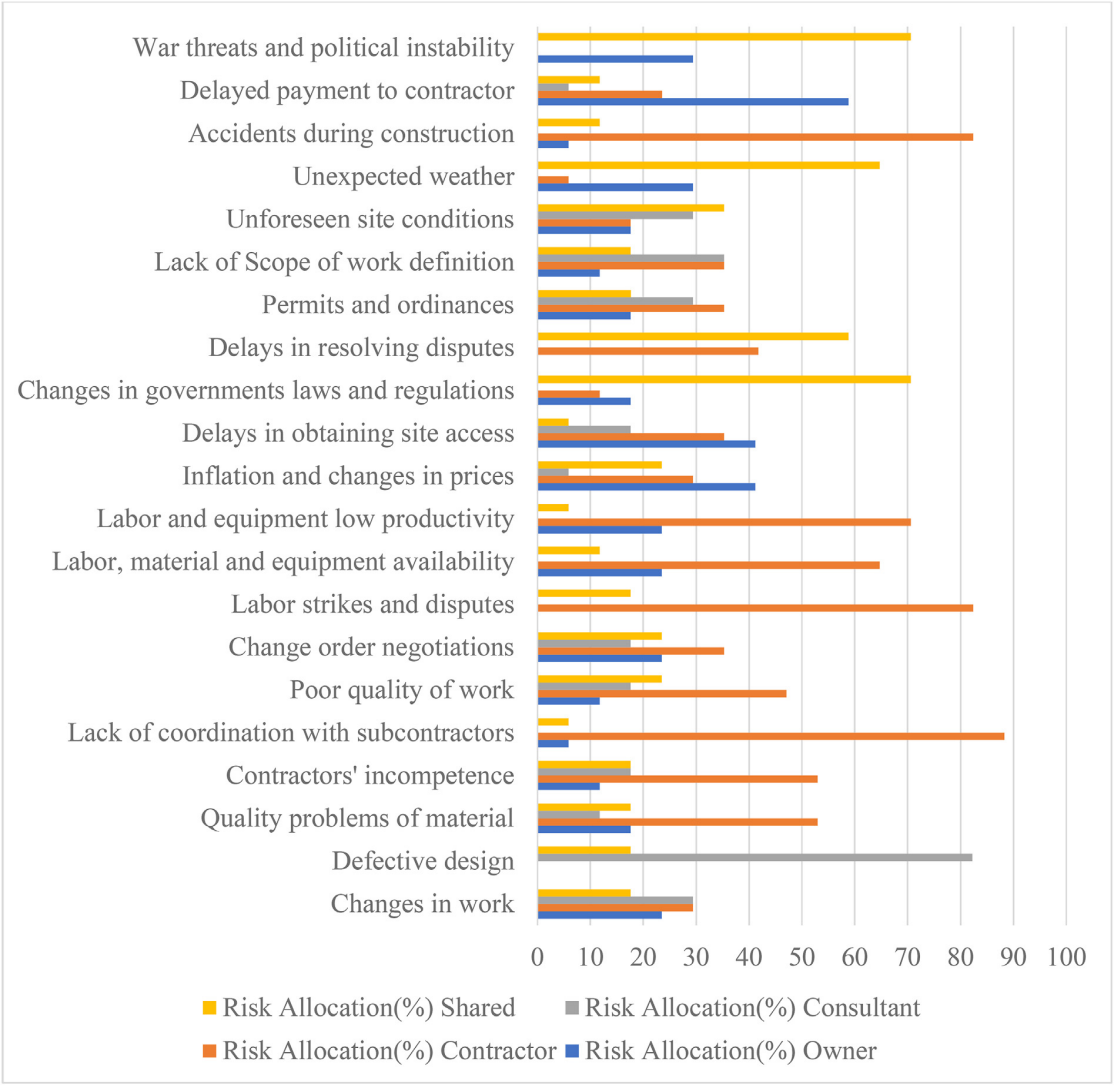
Bar chart Analysis of risk allocation.

The process of recognizing risk and choosing how and to what degree it should be shared is known as risk allocation, as shown in Figure 3. The majority of owners recognize that risk is an unavoidable aspect of the construction process. Many owners are unaware that by allocating risks to the right members of the construction team - the contractor, designer, or owner - the cost of risk management, and hence the project's construction expenses, can be decreased [43]. The risk allocation is determined by using Eq. (1) and the significance levels are shown in Table 2.

(1) $$Risk\;allocation\;\left(\%\right)=\frac{No\;of\;response\;to\;Questionnaire\;Survey}{No\;of\;Questionnaire\;Analyzed}\times 100$$

**Table 2:** Risk allocation to the shareholders in gondar city, Ethiopia.

Risk ID	Risk Factors	Risk Allocation (%)			
		Owner	Contractor	Consultant	Shared
R1	Changes in work	23.53	29.41	29.41	17.64
R2	Defective design	0.00	0.00	82.23	17.64
R3	Quality problems of material	17.64	52.96	11.76	17.64
R4	Contractors' incompetence	11.76	52.96	17.64	17.64
R5	Lack of coordination with subcontractors	5.88	88.24	0.00	5.88
R6	Poor quality of work	11.76	47.07	17.64	23.53
R7	Change order negotiations	23.53	35.30	17.64	23.53
R8	Labor strikes and disputes	0.00	82.36	0.00	17.64
R9	Labor, material and equipment availability	23.53	64.70	0.00	11.76
R10	Labor and equipment low productivity	23.53	70.58	0.00	5.88
R11	Inflation and changes in prices	41.17	29.411	5.88	23.53
R12	Delays in obtaining site access	41.17	35.29	17.64	5.88
R13	Changes in governments laws and regulations	17.64	11.76	0.00	70.60
R14	Delays in resolving disputes	0.00	41.764	0.00	58.82
R15	Permits and ordinances	17.64	35.29	29.41	17.66
R16	Lack of Scope of work definition	11.76	35.29	35.29	17.64
R17	Unforeseen site conditions	17.64	17.64	29.41	35.30
R18	Unexpected weather	29.41	5.88	0.00	64.70
R19	Accidents during construction	5.88	82.36	0.00	11.76
R20	Delayed payment to contractor	58.82	23.56	5.88	11.76
R21	War threats and political instability	29.41	0.00	0.00	70.59

Source: Authors (2022).

#### 2.6.2 Ranking of attributes

According to [44] the relative importance index (RII) approach is used to describe the relative importance of distinct factors and consequences using the 5-point Likert scale measurement instrument and the likelihood of occurrence and effect on the project. Furthermore, the critical cause or impact component is calculated by Eq. (2), and the larger value of the index of relative significance (RII) is the critical factor component.

The required information from the questionnaire survey is retrieved and graded using the quantitative method of the relative importance index. The RII approach is used to analyze the data. This method is used to generate scores for variables in order to assess and uncover hierarchical risk factors, and it uses a five-point Likert scale to determine the importance of each element [45]. The RII value ranges from zero (not inclusive) to one. Low (0 < RII< 0.8), and high level (0.8 < RII<1.0). Following the analysis, the various hazards were graded according to the severity of their influence on the construction project. For the organization and analysis data collected from the stakeholders Microsoft Excel was used as a tool. The relative importance index (RII) was determined as follows:

(2) $$RII=\frac{\sum W}{AN}=\frac{\sum W}{5N}$$

Where, w = Weight allotted to each risk (by response to questionnaire survey).

A = Highest weight (A = 5 for five-point Likert scale).

N = Total number of people (responses) who completed Questionnaire survey.

The attributes are ranked from highest to lowest impact on construction risk, with rank 1 having the most impact on construction performance. The attributes are arranged in ascending order of rank. However, RII doesn't make about the relationship between the various variables. The Spearman rank correlation is used to determine whether there is a relationship between the chosen attributes [46]. It evaluates how well a monotonic function can capture the relationship between two variables. The direction of the link between X and Y is indicated by the sign of the Spearman correlation. According to a zero Spearman correlation, there is no tendency for Y to either rise or decrease as X increases.

#### 2.6.3 Factor analysis

Factor analysis is a powerful statistical method that seeks to shed more light on the few fundamental factors that underlie many connected but seemingly unrelated variables [47, 48]. Data were entered using Microsoft Excel and exported to SPSS version 23 for factor analysis and regression analysis. The Kaiser-Meyer-Olkin (KMO) is calculated to measure the sampling adequacy, which indicates whether the sample size is big enough for conducting research or not. The Kaiser-Meyer-Olkin (KMO) test and Bartlett's test of sphericity [46]were used to determine whether the survey data were adequate for factor analysis. The ratio of the squared correlation between variables to the squared partial correlation between variables is represented by the value of KMO. It ranges from 0% to 100%. A number close 100% suggests that the pattern of correlations is relatively tight, and factor analysis should produce clear and trustworthy conclusions [46]. A suggested minimum value is 50% [49].

#### 2.6.4 Regression analysis

Linear multiple regression is used to further evaluate the factors that contribute to hazards in order to create a prediction model for the Ethiopian construction industry. Component analysis demonstrates the existence of clusters with high correlation coefficients and quantifiable underlying dimensions, but these components have minimal predictive power for the occurrences under consideration. On the other hand, the shared variance (value of R^2^ in the correlation matrix) is not taken into account by multiple regression analysis, which instead integrates a number of independent variables throughout the entire dataset [46]. The reliability of the link between the dependent or predictive variable and the independent variables can be determined by the value of R^2^, which is a reliable indicator. Several of the essential underlying assumptions in the predictor variables should be satisfied prior to doing the regression analysis. By examining the homogeneity of variance (Levene's test) on the chosen attributes, the parametric test was carried out in this study prior to the regression analysis [46]. Thus, the regression model constructed to assess the total influence of project performance caused by specific qualities can be determined in Eq. (3) as follows:

(3) $$Y=a+b_{1}x_{1}+b_{2}x_{2}+\ldots +b_{n}x_{n}\pm e$$

where Y is the risk factor variable, a is a constant, b_1_ to b_n_ are estimated regression coefficients, x_1_ to x_n_ are predictor or independent variable values, and e is the misclosure error.

## 3 Results and discussion

### 3.1 Risk allocation and ranking of attributes

**Figure 4:**
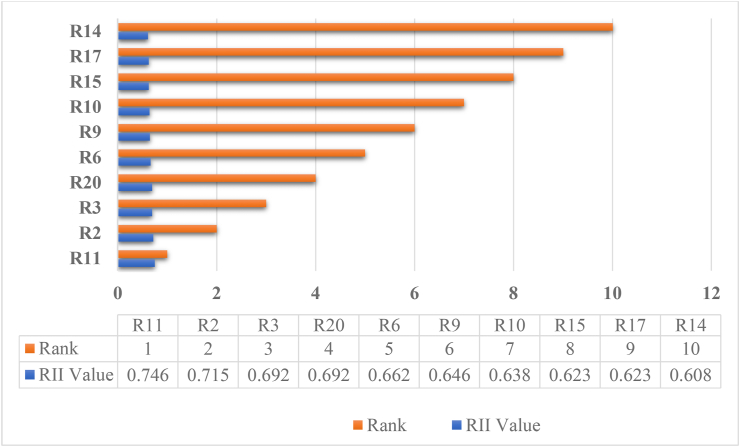
Bar chart Analysis of top harmful five obtained major risks.

**Figure 5:**
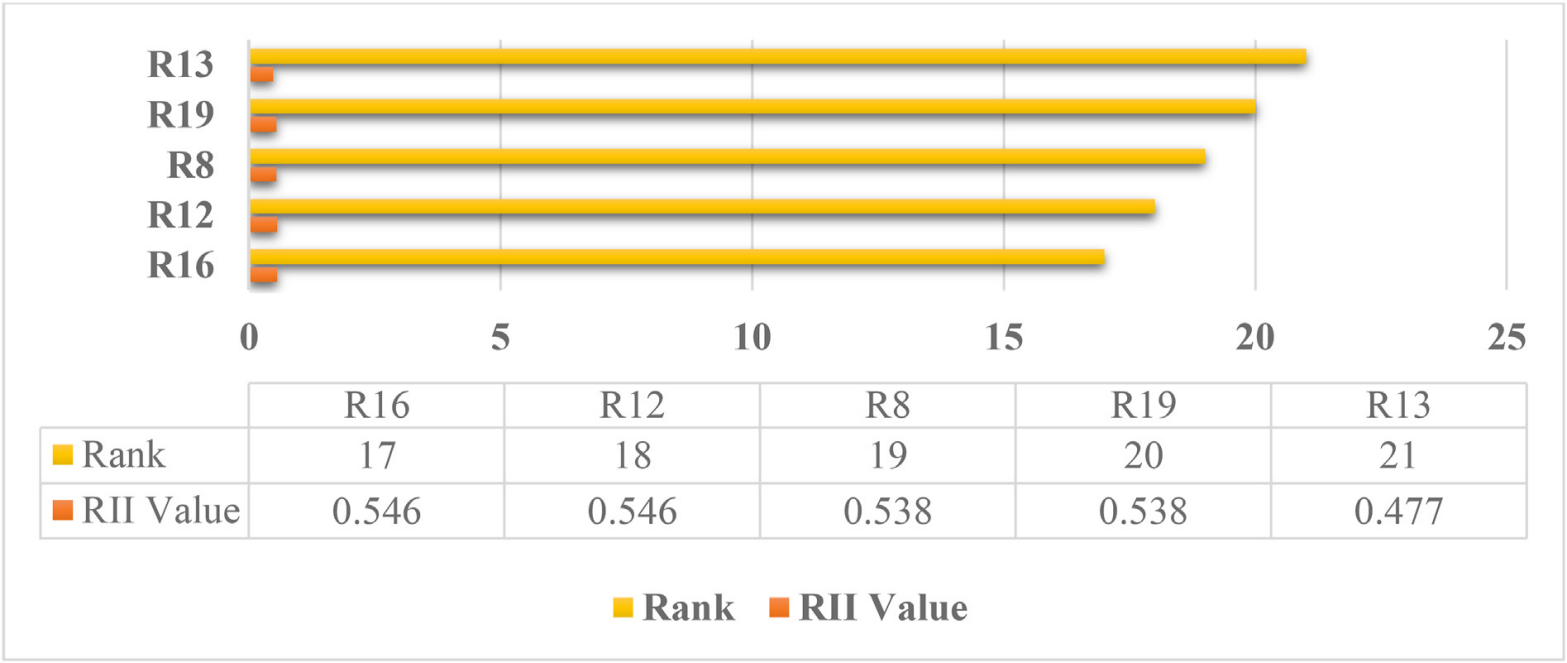
Bar chart Analysis of least harmful five obtained major risks.

The risk ranks and importance are attained from the analysis done by using Relative Importance Index (RII) method, based on the obtained responses via the Questionnaire survey. The ranks of the risks are based on how much harm they can cause to the project. The importance of the risks is based on the obtained ranks. In this analysis, according to 21 risk factors survey, the obtained top ten major risk analysis are R11, R2, R3, R20, R6, R9, R10, R15, R17, and R14 respectively shown in Figure 4 and the five least harmful risks to the building project analysis are R16, R12, R8, R19 and R13 respectively, shown in Figure 5.

Table 3 illustrated shows that the most risk factors and it reveals that the primary risk factors that influence construction projects in this study. As the findings delayed payment to contractor, inflation and changes in prices, and delays in obtaining site access are the severity of overall risk significance and allocation for client perspectives, lack of coordination, labor strikes and disputes, and accident during construction for the severity risk factor for contractor perspectives. Whereas, defective design, lack of scope of work, changes in work, permits and ordinates, and unforeseen site condition the severity risk factor for consultant perspectives and for the shared perspectives severity risk factor are also changes in government law and regulations, war threats and political instability, and unexpected weather. According to [30], the top risk factors faced by the construction industry parties (client and contractor perspectives) are awarded design to unqualified designers, accepted defective design, occurrence of accidents because of poor safety procedures, difficulty to access the site, inaccurate quantities, lack of consistency between bill of quantities, drawing and specification, working a hot (dangerous) area, financial failure of the contractor, and high competition in bids. Inflation and changes in prices, defective design and quality problems of material respectively under economic, and construction and design categories are the three common risk factors in Gondar city. It is evident that respondents believe these factors have a high impact on construction projects with a value of RII 0.746, 0.715 and 0.692 respectively. According to [50] the top risk factors are as delay in client decision making and error in estimated cost and construction period. According to [51], the primary risk elements in home construction projects in Kathmandu are turnaround time, scope of project hazards, financial and economic risks, organizational risks, safety and health risks, and communication risks. Changes in scope may occur as projects proceed from design to completion, according to the questionnaire performed. Project managers or design teams are usually the ones who change the scope. As a result, a project could fail due to a lack of communication and ambiguity [52]. In general, project owners' lack of delays in project service payment can cause project activities to be delayed or even terminated [53]. As the findings, in this paper the top five major risks showed that 40% of the risk to the construction project is from the owner's perspective, another 40% of the risk is from the contractor's perspective, and the remaining 20% of the risk is from the consultant's perspective. Whereas, from results the top five major risk factors, 60% of the risk is from the construction and design risk, 20% of the risk is from the economic risk, and the remaining 20% of the risk is from other risk categories.

**Table 3:** Ranking of attributes/factors.

Risk category	Risk ID	Risk Factors	RII	Rank	Importance level
Construction and Design	R1	Changes in work	0.569	14	Medium
	R2	Defective design	0.715	2	High-medium
	R3	Quality problems of material	0.692	3	High-medium
	R4	Contractors' incompetence	0.577	13	Medium
	R5	Lack of coordination with subcontractors	0.562	16	Medium
	R6	Poor quality of work	0.662	5	High-medium
Management	R7	Change order negotiations	0.585	11	Medium
	R8	Labor strikes and disputes	0.538	19	Medium
Finance	R9	Labor, material and equipment availability	0.646	6	High-medium
	R10	Labor and equipment low productivity	0.638	7	High-medium
Economic	R11	Inflation and changes in prices	0.746	1	High-medium
Political	R12	Delays in obtaining site access	0.546	18	Medium
	R13	Changes in governments laws and regulations	0.477	21	Medium
	R14	Delays in resolving disputes	0.608	10	High-medium
Law and order	R15	Permits and ordinances	0.623	8	High-medium
Technical	R16	Lack of Scope of work definition	0.546	17	Medium
Climate Condition	R17	Unforeseen site conditions	0.623	9	High-medium
	R18	Unexpected weather	0.577	12	Medium
Accidental	R19	Accidents during construction	0.538	20	Medium
Others	R20	Delayed payment to contractor	0.692	4	High-medium
	R21	War threats and political instability	0.569	15	Medium

Source: Authors (2022).

According to previous research focused on analysis of major risks in construction projects, the top five major risks in the construction project are environmental risks, design risks, financial risks, physical risks, and market risks [54]. This showed that the ranking risk in construction projects varies from one country to another. In India, environmental risks are the dominant risk factor, but in Gondar, Ethiopia, environmental factors are not even in the top ten risk factors. And the result showed that the defective design factor is almost the predominant risk factor everywhere.

According to [55], the finding showed that inadequate schedule, nonpayment and minimum amount of interim payment and submittals and approvals of construction documents is the first, second and the third ranked most critical risk in the Ethiopian construction industry, with an RII value of 0.900 and both with an RII value of 0.878 respectively. The finding showed that construction and design risks, and economic risks are the most critical risks in Gondar city, Ethiopian construction industry.

### 3.2 Factor analysis

The analysis of the 21 attributes based on association in this study is limited to 17 attributes in total. The remaining four were excluded in the study because it was found that there was no meaningful relationship between them. The KMO value of 56% for the 17 variables included in this study is thought to be good. Principal component analysis is used to condense a large number of associated attribute variables into a much smaller set of underlying factors using the retrieved components. Eight (8) major components (factors) in all were created, and the outcomes are listed in Table 4. 81.80% of the total variance was explained by these eight variables. The results on the orthogonal factors can be better interpreted thanks to the Varimax rotation.

**Table 4:** Summary of Factor Analysis from extracted components.

ID	Factor Classification	Factor Description	Factor Loading	% Variance explained
R3	1) Construction and Design	Quality problems of material	0.796	18.851
R6		Poor quality of work	0.633	
R5		Lack of coordination with subcontractors	0.576	
R4		Contractors' incompetence	0.523	
R2		Defective design	0.504	
R8	2) Management	Labor strikes and disputes	0.663	10.919
R7		Change order negotiations	0.541	
R9	3) Finance	Labor, material and equipment availability	0.689	9.663
R10		Labor and equipment low productivity	0.640	
R14	4) Political	Delays in resolving disputes	0.710	9.476
R12		Delays in obtaining site access	0.578	
R11	5) Economic	Inflation and changes in prices	0.560	8.821
R15	6) Law and Order	Permits and ordinances	0.728	8.185
R18	7) Climate Conditions	Unexpected weather	0.766	8.091
R17		Unforeseen site conditions	0.657	
R20	8) Others	Delayed payment to contractor	0.695	7.789
R21		War threats and political instability	0.695	

Table 4 displays the extracted components. The first element, risk of construction and design, accounts for 18.85% of the variation of the linear component's five attributes. The second factor, management risk, explains that two important characteristics account for 10.92% of the total variance of the linear component. The presence of two qualities in 9.66% of the total variation of the linear component is explained by the third factor, financial risk. Political issues, the fourth factor, explain that the underpinning characteristics accounting for 9.48% of the total variance are two. The fifth factor, lack of economics in the project, reveals that only one attribute (inflation and changes in prices) accounts for around 8.82% of the total variation of the linear component and has a factor loading of 0.56. The sixth factor, a lack of law and order, only has one attribute yet is responsible for 8.19% of the variation. Climate conditions and other dangers, which make up the seventh and eighth components, respectively, account for 8.09% and 7.79% of the total variance for these two crucial characteristics. The cumulative variance explained by the eight extracted components is 81.80%, which is thought to be an excellent figure for the best factor analysis outcomes. According to the findings of the factor analysis, there are eight main components that account for 81.80% of the variance in the risk factors, which is more than the 79.83% of total variance for risk factors advised by [56].

### 3.3 Regression analysis

The regression model is created using a methodical, sequential process. Although it might not be the best model, Spearman correlation is used to find predictor factors that strongly correlate with the dependent variable. Then, a stepwise forward-backward technique and forced entrance are used to construct new models. Updated R^2^ values are a good sign of a robust model since they show how the value of R^2^ evolves as more independent variables are added. The adjusted R^2^ values and the change from R^2^ values demonstrate the model's generalization capability of the dependent variable's predictive power [48]. In a perfect scenario, R^2^ and adjusted R^2^ values should coincide. The strength of the model's prediction is determined by the difference between R^2^ and adjusted R^2^; the smaller the difference, the more potent the model [46]. The values reported in Table 5 are acceptable, with tolerable strengths based on the goodness of the model fit.

**Table 5:** Regression model analysis.

Model	Constant and Coefficient	Std Error	Prob.	R^2^ and adjusted R^2^
Constant	-0.507	3.426	-	R^2^ = 0.991, adjusted R^2^ = 0.940, F = 19.530, p = 0.016, Dublin-Watson = 1.522
R11	1.083	0.109	0.002	
R12	0.313	0.099	0.050	
R20	0.524	0.134	0.030	

The final regression model for impact of risk can be substituted in Eq. (3) expressed as:

$$Y=-0.507+1.{083x}_{1}+0.313x_{2}+0.524x_{3}$$

where Y is the impact of risk factors, -0.507 is a constant, 1.083, 0.313 and 0.524 are estimated regression coefficients, and x_1_, x_2_ and x_3_ are inflation and changes in prices, delays in obtaining site access and delayed payment to contractor respectively predictor variable values.

The regression model makes it abundantly evident that inflation and changes in prices from economic (R11), delays in obtaining site access from political aspects (R12) and delayed payment to contractor from other risk classification (R20) have the greatest influence on the extent of risks in Ethiopian construction projects. These findings appear to have similarities to the findings of [57, 58] about the synergy between practices in developing nations. When the descriptive rating of the features in Table 3 is compared with the regression model's conclusions mentioned above, a stark disparity between the attributes' perceived importance and how much they affect risk factor is evident. Comparing the top five attributes reported in Table 3, two of them (R11&R20) were found to be significant in terms of in terms of predicting risks in construction projects in the context of Gondar, Ethiopia. In the regression model, attributes R12, which is ranked eighteenth have considerable predictive potential. All three attributes in the regression model show a clear contribution in terms of achieving performance in Gondar, Ethiopian construction projects. Such a disparity draws attention to the fact that, although descriptive analysis is helpful and instructive, its criticality in terms of its linkages with the phenomena being measured needs to be further assessed using the appropriate statistical procedures [46, 59].

## 4 Conclusion

In Ethiopia's construction industry, the idea of sustainable construction is still relatively new. Due to a number of obstacles, including the risks involved, its degree of adoption in the building sector has also been determined to be very low. Hence, there is a need for insight into the degree of risk associated with applying sustainable construction methods in Ethiopia's construction sector. The primary aim of this paper was to evaluate the impact of construction risk on project performance. Questionnaire survey using Likert-scale was used to collect the responses from the respondents. 26 valid responses collected from the construction professionals were used to analyses the data.

According to RII, the findings showed that the major risks are: inflation and changes in prices; defective design; quality problems of materials; delayed payment to the contractor; poor quality of work, labor material and equipment availability; labor and equipment low productivity; permits and ordinances; unforeseen site conditions; and delays in resolving disputes. According to the findings, the majority of the risk classifications are economic risks, construction and design risks, other risks, financial risks, laws and order risks, climate condition risks, and political risks. The study's limitation is that it is limited to risk factors on construction projects in Gondar city, Ethiopia. This implied that the idea of sustainable construction was not well established in Ethiopia's building construction sector. Therefore, stakeholders in the building business need to be more aware of sustainable construction. This could be accomplished through instruction and training, teamwork and knowledge sharing, and other forms of communication. More study is required to create new sustainable building materials and make them accessible and inexpensive, according to this as well. In addition, it is crucial to have the right legal and socioeconomic frameworks in place to direct the adoption of sustainable building techniques. Using component extracted factor analysis, the 18 variables of construction projects were recovered, and 17 independent factors (risk factors) and 1 dependent factor (project performance) were extracted. The factor analysis's findings indicate that [56] satisfies the criteria that there be eight basic components, which together account for 81.80% of the variance in the risk factors. As a result, the regression model's examination of additional risk factors reveals that inflation and changes in prices, delays in obtaining site access and delayed payment to contractor payments all significantly impact the success of construction projects in Gondar, Ethiopia. In general, the top five risk attributes in all data analysis scenarios were inflation, changes in pricing, and delayed payment to contractors due to other risk attributes, according to an examination of the effects of collective risk factors on performance indicators in building projects.

### 4.1 Limitations and scope for future study

Even if the findings of this study have a significant impact on the corporate sector, there are numerous limitations. A sample size of 26 is first considered to be too small for statistical analysis. Second, the results may have been skewed due to the respondents' unequal distribution among the professional roles. As a result, the developed model may be improved further based on in-depth conversations and recommendations from industry experts. It is predicted that the author's future work will delve deeper into the connections between various risk factors and how they affect project risk as a whole. As part of the final recommendations, the Ethiopian Construction Management Institute (ECMI) and other building material research groups were asked to step up their efforts to develop creative, affordable, and sustainable building materials. Another recommendation was the creation of an appropriate legal framework for the administration of sustainable construction projects. As a result, additional studies might be carried out using infrastructure projects related to civil engineering (dams, roads, bridges, etc.).

## Declarations

### Author contribution statement

Amare Tilahun: Conceived and designed the experiments; Performed the experiments; Analyzed and interpreted the data.

Getachew Asefa: Performed the experiments; Analyzed and interpreted the data.

Natnael Melsew: Analyzed and interpreted the data; Wrote the paper.

### Funding statement

This research did not receive any specific grant from funding agencies in the public, commercial, or not-for-profit sectors.

### Data availability statement

Data will be made available on request.

### Declaration of interest's statement

The authors declare no conflict of interest.

### Additional information

No additional information is available for this paper.
